# Supplementary material for: dCas13-mediated translational repression for accurate gene silencing in mammalian cells
Source: Nat Commun. 2024 Mar 11;15:2205. doi: 10.1038/s41467-024-46412-7 (PMC10928199; doi:10.1038/s41467-024-46412-7)
Supplement: Supplementary file 1 — Supplementary Information [file 41467_2024_46412_MOESM1_ESM.pdf]

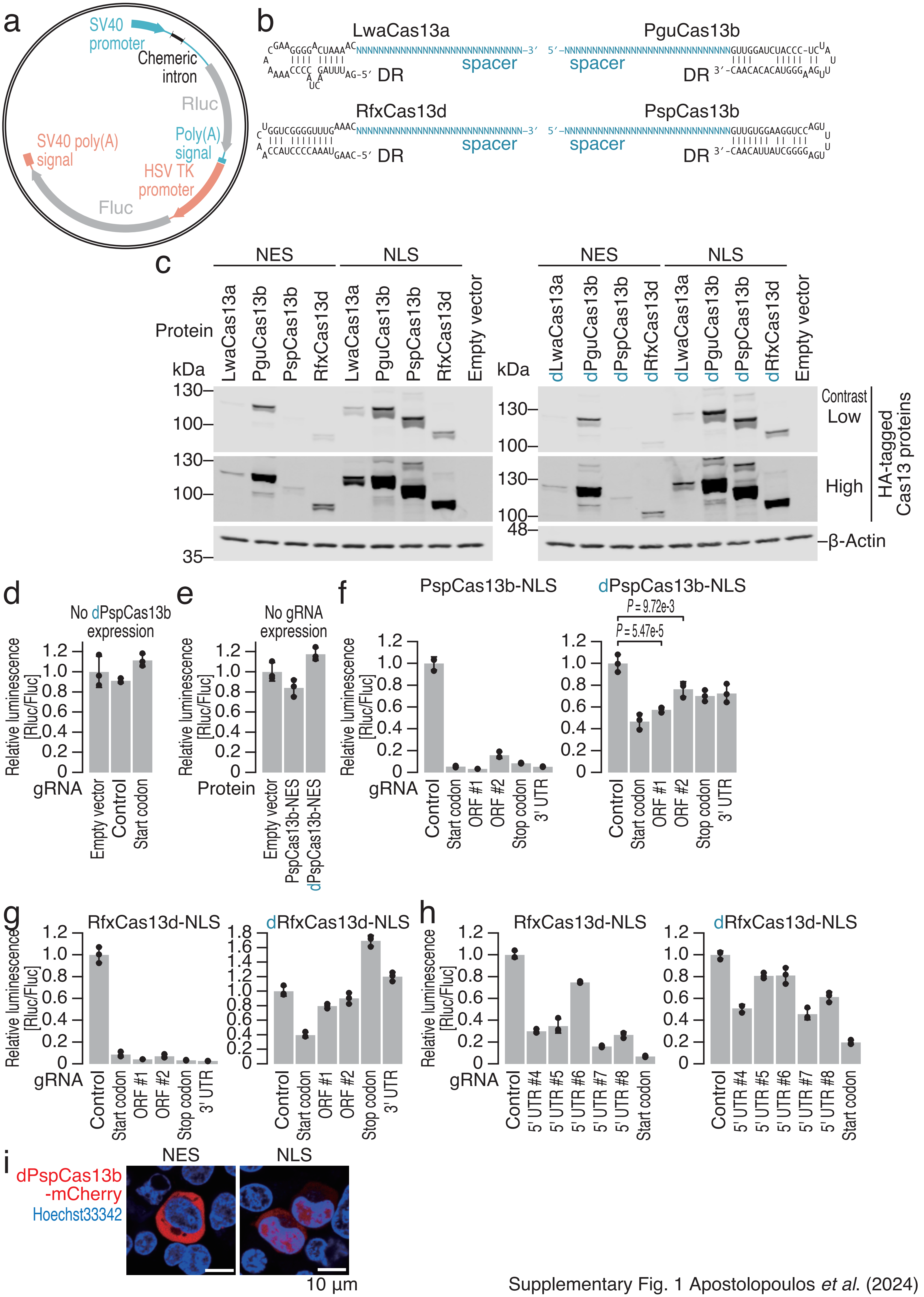

Supplementary Fig. 1 Apostolopoulos *et al.* (2024)

Supplementary Fig. 1: Characterization of the potency of the Cas13 protein for translational repression.

(a) Schematic of the plasmid used for the dual-reporter assay.

(b) Schematic of the gRNA architecture for each Cas13 protein.

(c) Western blotting of the indicated proteins. β-Actin was used as a loading control. Low- and high-contrast images of the anti-HA blot are shown.

(d and e) Relative Rluc luminescence with respect to Fluc luminescence was calculated to quantify the repressive effects of gRNA alone (d) and of dPspCas13b-NES alone (e).

(f) Relative Rluc luminescence with respect to Fluc luminescence was calculated to quantify the repressive effects of PspCas13b-NLS (left) and dPspCas13b-NLS (right) using the gRNAs shown in Fig. 1e.

(g) Relative Rluc luminescence with respect to Fluc luminescence was calculated to quantify the repressive effects of RfxCas13d-NLS (left) and dRfxCas13d-NLS (right) using the gRNAs shown in Fig. 1e.

(h) Relative Rluc luminescence with respect to Fluc luminescence was calculated to quantify the repressive effects of RfxCas13d-NLS (left) and dRfxCas13d-NLS (right) using the gRNAs shown in Fig. 1g.

(i) Microscopy image of mCherry-tagged dPspCas13 variants with an NES or NLS. DNA was stained with Hoechst 33342. Scale bar, 10 μm.

In d-h, the mean (gray bar), s.d. (black line), and individual replicates (n = 3, black points) are shown. In f, the p values were calculated by the Tukey–Kramer test (two-tailed).

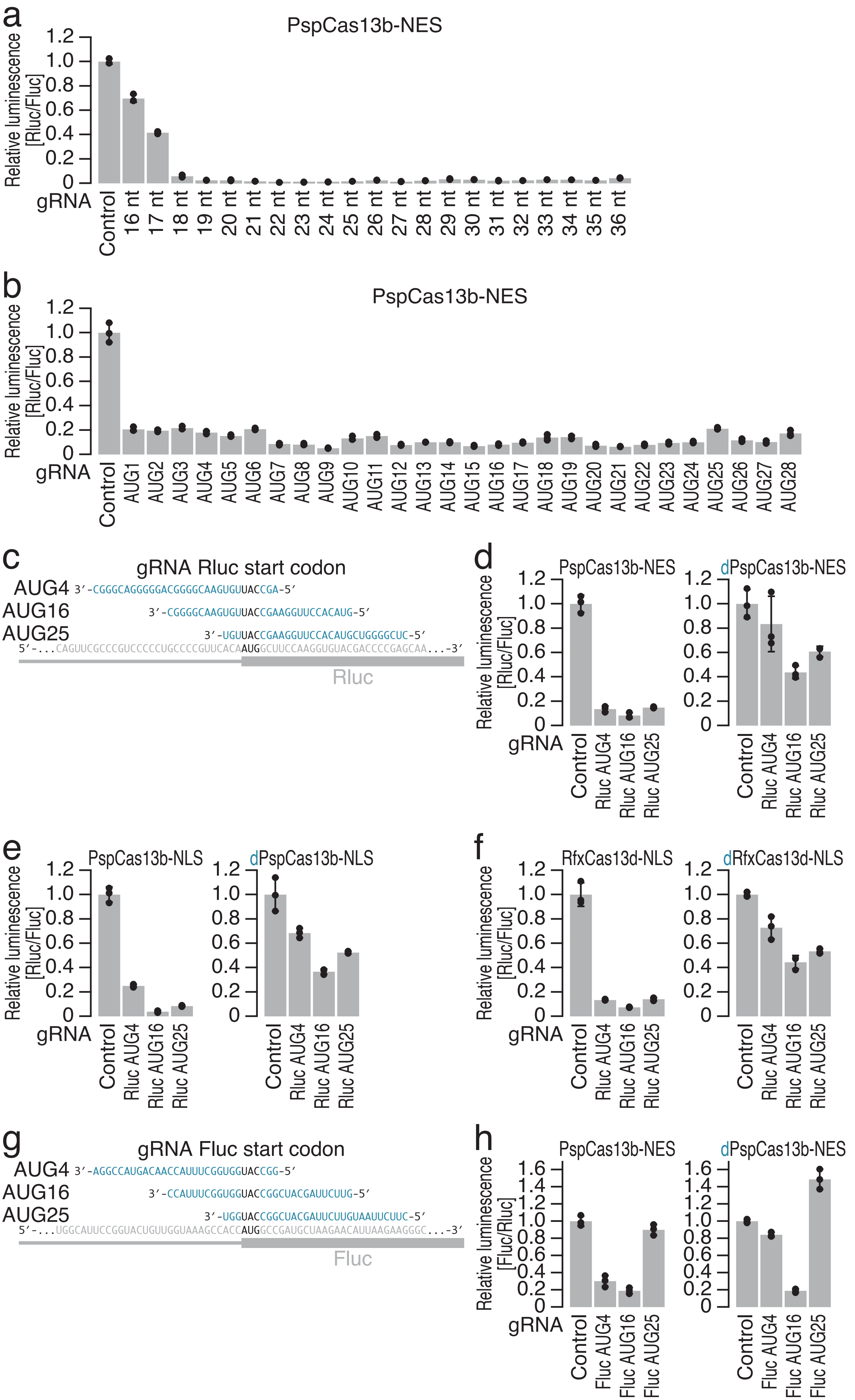

Supplementary Fig. 2 Apostolopoulos *et al.* (2024)

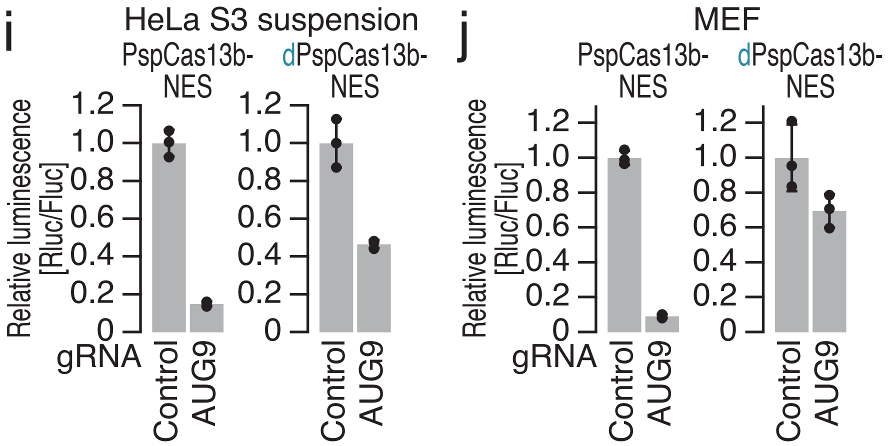
i
HeLa S3 suspension
PspCas13b-NES
dPspCas13b-NES
Relative luminescence [Rluc/Fluc]
1.2
1.0
0.8
0.6
0.4
0.2
0
gRNA
Control
AUG9
j
MEF
PspCas13b-NES
dPspCas13b-NES

Supplementary Fig. 2: Characterization of gRNA potency for translational repression.

(a and b) Relative Rluc luminescence with respect to Fluc luminescence was calculated to quantify the repressive effects of PspCas13b-NES using the gRNAs shown in Fig. 2a, c.

(c and g) Schematic of the gRNA design for targeting the start codon of Rluc reporter mRNA (c) and Fluc reporter mRNA (g).

(d) Relative Rluc luminescence with respect to Fluc luminescence was calculated to quantify the repressive effects of PspCas13b-NES (left) and dPspCas13b-NES (right) using the gRNAs shown in c.

(e) Relative Rluc luminescence with respect to Fluc luminescence was calculated to quantify the repressive effects of PspCas13b-NLS (left) and dPspCas13b-NLS (right) using the gRNAs shown in c.

(f) Relative Rluc luminescence with respect to Fluc luminescence was calculated to quantify the repressive effects of RfxCas13d-NLS (left) and dRfxCas13d-NLS (right) using the gRNAs shown in c.

(h) Relative Fluc luminescence with respect to Rluc luminescence was calculated to quantify the repressive effects of PspCas13b-NES (left) and dPspCas13b-NES (right) using the gRNAs shown in g.

(i and j) Relative Rluc luminescence with respect to Fluc luminescence was calculated to quantify the repressive effects of PspCas13b-NES (left) and dPspCas13b-NES (right) using the gRNAs shown in Fig. 2c. The indicated cells were used.

In a, b, d-f, and h-j, the mean (gray bar), s.d. (black line), and individual replicates (n = 3, black points) are shown. In a, b, d-f, and h, HEK293 cells were used.

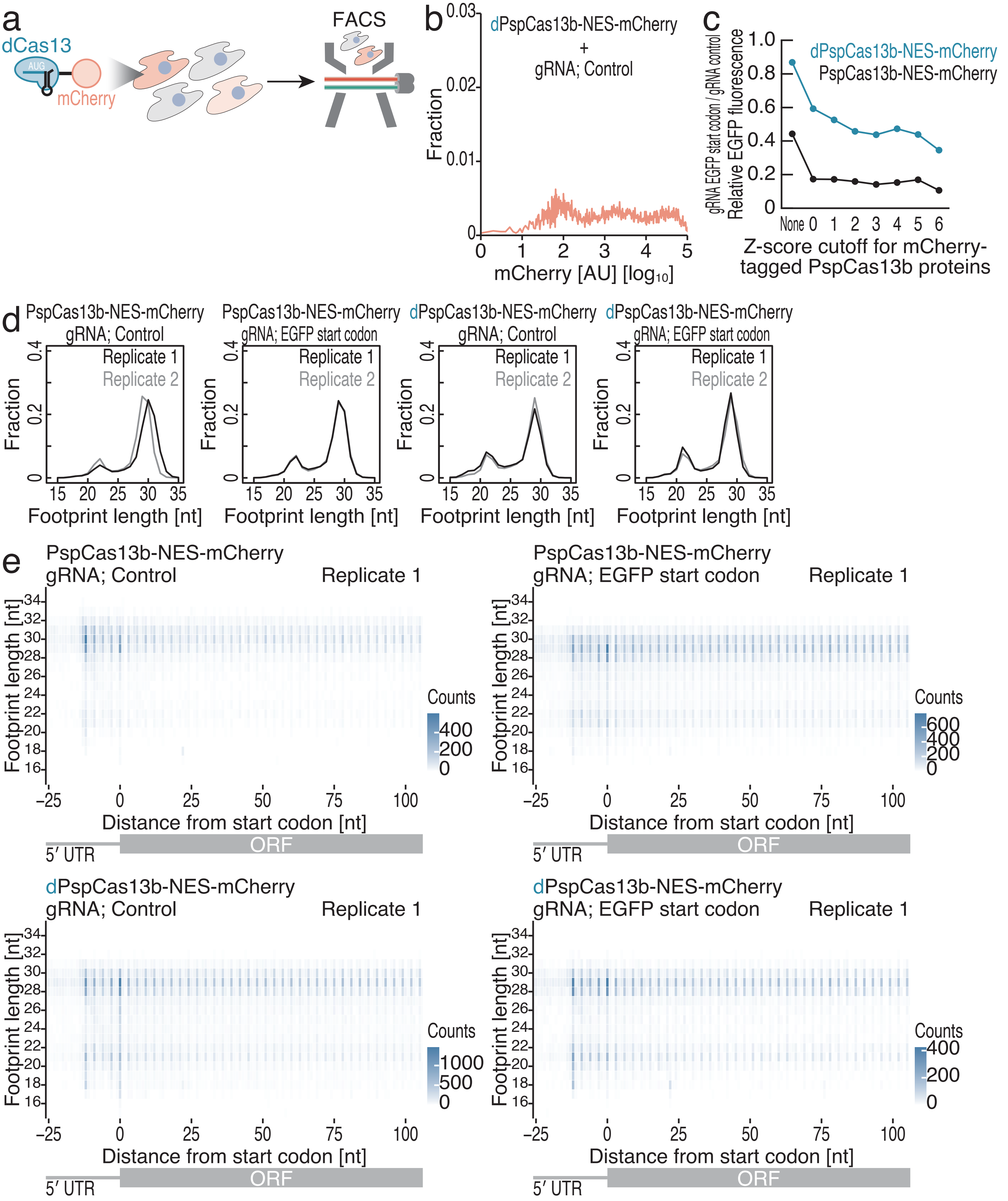
a
dCas13
AUG
mCherry
FACS
b
dPspCas13b-NES-mCherry
+
gRNA; Control
Fraction
mCherry [AU] [log10]
c
dPspCas13b-NES-mCherry
PspCas13b-NES-mCherry
gRNA EGFP start codon / gRNA control
Relative EGFP fluorescence
Z-score cutoff for mCherry-tagged PspCas13b proteins
None
d
PspCas13b-NES-mCherry
gRNA; Control
PspCas13b-NES-mCherry
gRNA; EGFP start codon
dPspCas13b-NES-mCherry
gRNA; Control
dPspCas13b-NES-mCherry
gRNA; EGFP start codon
Replicate 1
Replicate 2
Fraction
Footprint length [nt]
e
PspCas13b-NES-mCherry
gRNA; Control
Replicate 1
PspCas13b-NES-mCherry
gRNA; EGFP start codon
Replicate 1
dPspCas13b-NES-mCherry
gRNA; Control
Replicate 1
dPspCas13b-NES-mCherry
gRNA; EGFP start codon
Replicate 1
Footprint length [nt]
Counts
Distance from start codon [nt]
5′ UTR
ORF

f

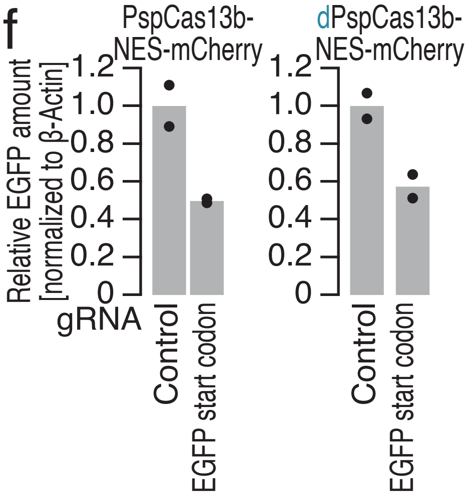
PspCas13b-
NES-mCherry
dPspCas13b-
NES-mCherry
Relative EGFP amount
[normalized to β-Actin]
1.2
1.0
0.8
0.6
0.4
0.2
0
gRNA
Control
EGFP start codon

Supplementary Fig. 3: Characterization of ribosome profiling data with flow cytometry-sorted cells.

(a) Schematic of the experimental procedure for measuring EGFP (reporter) and mCherry (PspCas13b variant) expression. PspCas13b variant expression was ensured by cell sorting based on mCherry expression.

(b) Representative distribution of the expression level of mCherry-fused dPspCas13b.

(c) Relative EGFP fluorescence plotted against the PspCas13b variant expression level. Signals from the fused mCherry were used to assess PspCas13b expression. Data from 1×10^4 cells were used for the analysis, with subpopulation means determined with the indicated Z score cutoffs.

(d) Footprint length distributions under the indicated conditions.

(e) Metagene plots for the 5′ ends of ribosome footprints for the indicated samples. The data were aligned to the start codons. The color scales for the reads are shown.

(f) Quantification of the Western blot signal for EGFP in Fig. 3e. The mean (gray bar) and individual replicates (n = 2, black points) are shown.

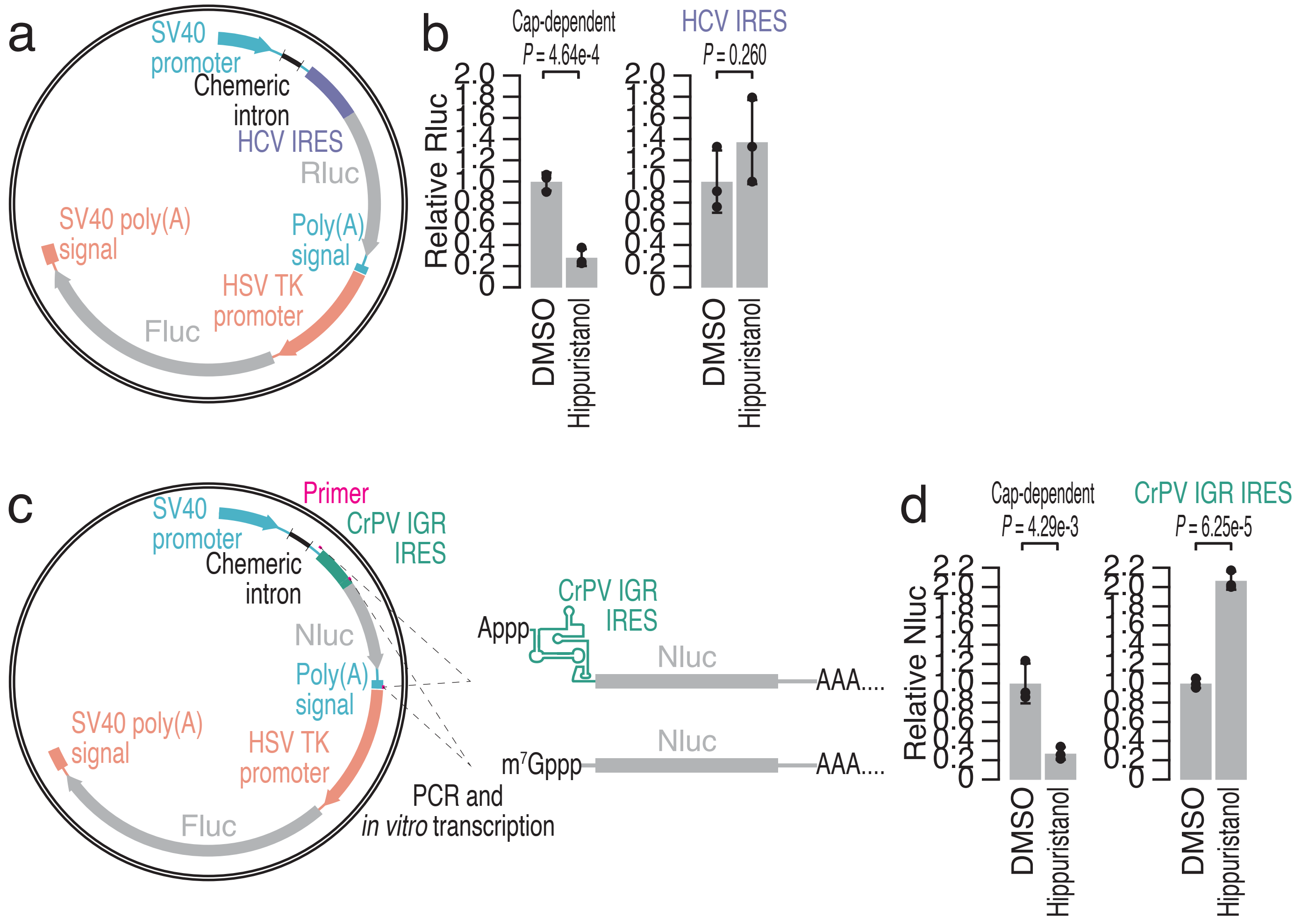

Supplementary Fig. 4 Apostolopoulos *et al.* (2024)

Supplementary Fig. 4: Characterization of HCV IRES-driven translation.

(a) Schematic of the HCV IRES-containing reporter plasmid.

(b and d) Relative Rluc or Nluc luminescence from the indicated reporter mRNAs after 1 μM hippuristanol treatment.

(c) Schematic of the CrPV IGR IRES-containing reporter plasmid used for in vitro transcription. Template DNA fragments for A-capped CrPV IGR IRES mRNA and m^7G-capped control mRNA were PCR-amplified by the indicated primer sets.

In b and d, the mean (gray bar), s.d. (black line), and individual replicates (n = 3, black points) are shown. In b and d, p values were determined by Student's t test (two-tailed).

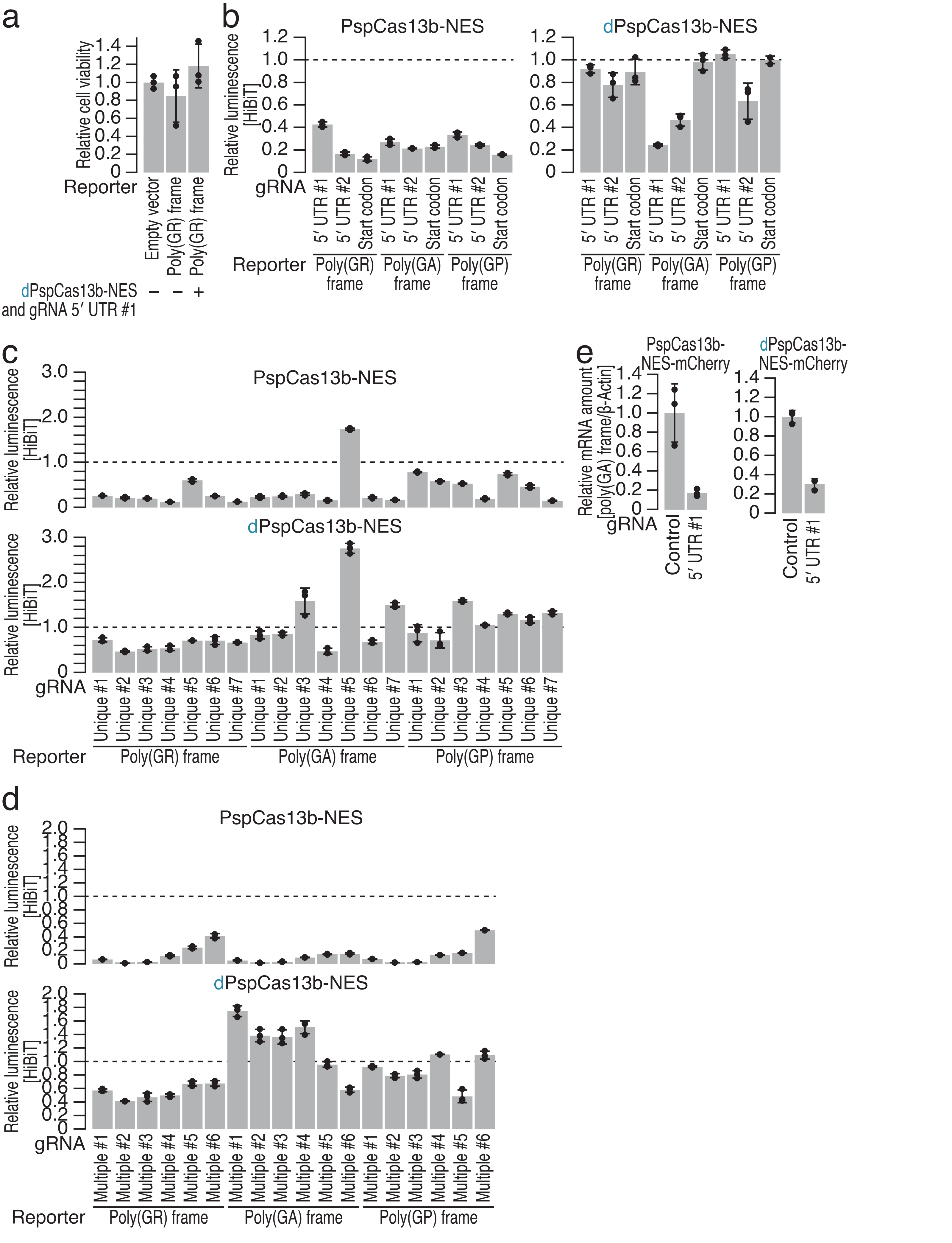
a
Relative cell viability
Reporter
Empty vector
Poly(GR) frame
Poly(GR) frame
dPspCas13b-NES and gRNA 5′ UTR #1
b
PspCas13b-NES
dPspCas13b-NES
Relative luminescence [HiBiT]
gRNA
5′ UTR #1
5′ UTR #2
Start codon
Reporter
Poly(GR) frame
Poly(GA) frame
Poly(GP) frame
c
PspCas13b-NES
dPspCas13b-NES
Relative luminescence [HiBiT]
gRNA
Unique #1
Unique #2
Unique #3
Unique #4
Unique #5
Unique #6
Unique #7
Reporter
Poly(GR) frame
Poly(GA) frame
Poly(GP) frame
d
PspCas13b-NES
dPspCas13b-NES
Relative luminescence [HiBiT]
gRNA
Multiple #1
Multiple #2
Multiple #3
Multiple #4
Multiple #5
Multiple #6
Reporter
Poly(GR) frame
Poly(GA) frame
Poly(GP) frame
e
PspCas13b-NES-mCherry
dPspCas13b-NES-mCherry
Relative mRNA amount [poly(GA) frame/β-Actin]
gRNA
Control
5′ UTR #1

Supplementary Fig. 5: Characterization of the ability of gRNA to reduce RAN translation.

(a) Relative cell viability according to the expression of GGGGCC repeat reporter mRNA [poly(GR) frame].

(b-d) Relative HiBiT luminescence was calculated to quantify the repressive effects of PspCas13b-NES or dPspCas13b-NES using the gRNAs shown in Fig. 5a. The same data were used for Fig. 5b-d.

(e) Relative GGGGCC repeat reporter [poly(GA) frame] abundance with respect to endogenous β-Actin mRNA abundance was quantified by RT–qPCR under the indicated conditions.

In a-e, the mean (gray bar), s.d. (black line), and individual replicates (n = 3, black points) are shown.

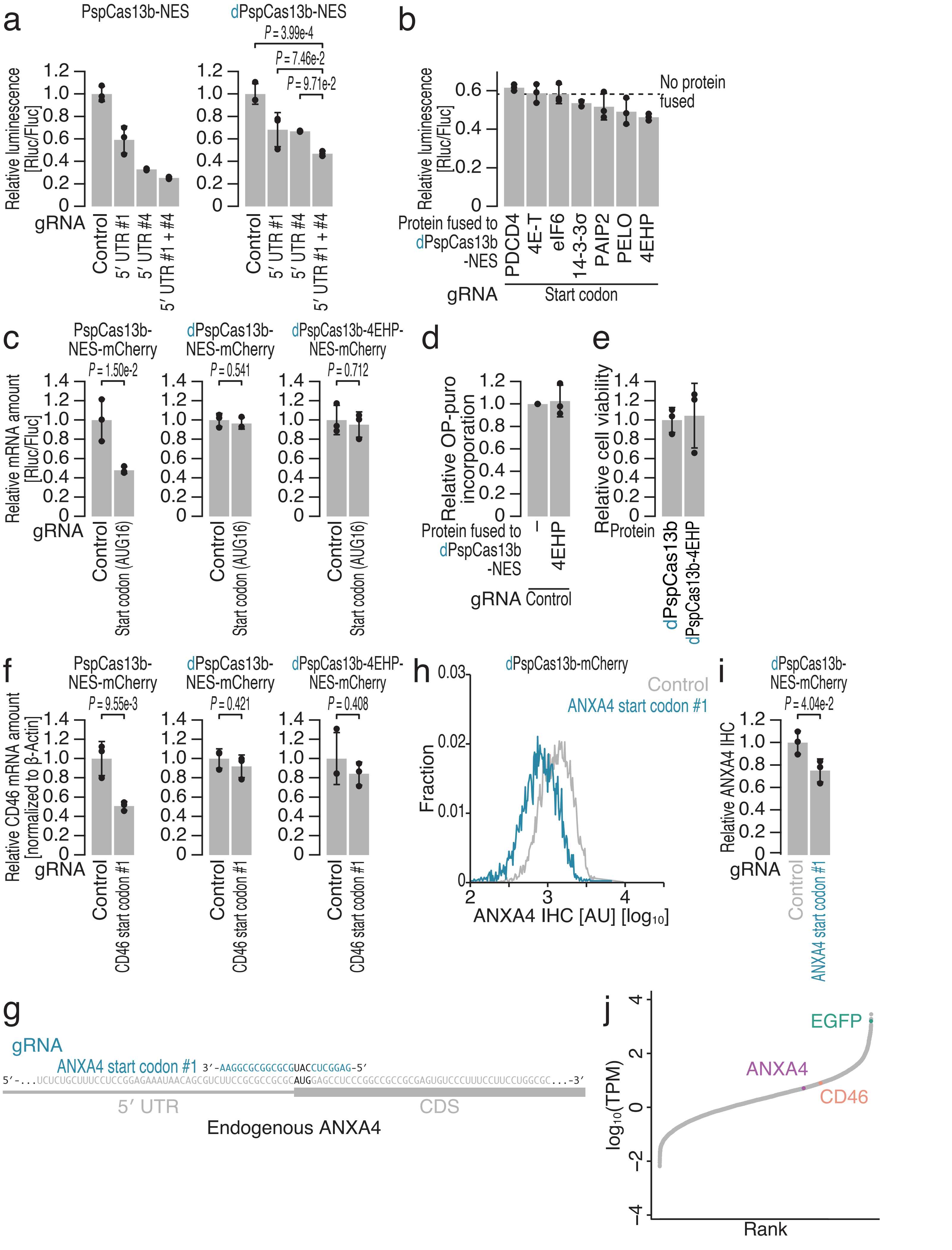
a
PspCas13b-NES
dPspCas13b-NES
P = 3.99e-4
P = 7.46e-2
P = 9.71e-2
Relative luminescence [Rluc/Fluc]
gRNA
Control
5′ UTR #1
5′ UTR #4
5′ UTR #1 + #4
b
No protein fused
Protein fused to dPspCas13b-NES
PDCD4
4E-T
eIF6
14-3-3σ
PAIP2
PELO
4EHP
Start codon
c
PspCas13b-NES-mCherry
dPspCas13b-NES-mCherry
dPspCas13b-4EHP-NES-mCherry
P = 1.50e-2
P = 0.541
P = 0.712
Relative mRNA amount [Rluc/Fluc]
Start codon (AUG16)
d
Relative OP-puro incorporation
–
4EHP
gRNA Control
e
Relative cell viability
Protein
dPspCas13b
dPspCas13b-4EHP
f
P = 9.55e-3
P = 0.421
P = 0.408
Relative CD46 mRNA amount [normalized to β-Actin]
CD46 start codon #1
h
dPspCas13b-mCherry
Control
ANXA4 start codon #1
Fraction
ANXA4 IHC [AU] [log10]
i
dPspCas13b-NES-mCherry
P = 4.04e-2
Relative ANXA4 IHC
ANXA4 start codon #1
g
gRNA
ANXA4 start codon #1 3′-AAGGCGCGGCGCGUACCUCGGAG-5′
5′-...UCUCUGCUUUCCUCCGGAGAAAUAACAGCGUCUUCCGCGCCGCGCAUGGAGCCUCCCGGCCGCCGCGAGUGUCCCUUUCCUUCCUGGCGC...-3′
5′ UTR
CDS
Endogenous ANXA4
j
EGFP
ANXA4
CD46
log10(TPM)
Rank

Supplementary Fig. 6: Options for improving CRISPRδ efficacy.

(a) Relative Rluc luminescence with respect to Fluc luminescence was calculated to quantify the repressive effects of PspCas13b-NES and dPspCas13b-NES using the gRNAs shown in Fig. 1g.

(b) Relative Rluc luminescence with respect to Fluc luminescence was calculated to quantify the repressive effects of dPspCas13b-NES alone and that fused to translational repressors using a gRNA targeting the start codon of the Rluc reporter mRNA.

(c) Relative Rluc mRNA abundance with respect to Fluc mRNA abundance was quantified by RT–qPCR under the indicated conditions.

(d) Relative levels of OP-puro-labeled proteins with respect to total proteins were calculated to quantify the change in global translation induced by dPspCas13b-4EHP-NES.

(e) Relative cell viability according to the expression of the indicated dPspCas13b variants.

(f) Relative endogenous CD46 mRNA abundance with respect to endogenous β-Actin mRNA abundance was quantified by RT–qPCR under the indicated conditions.

(g) Schematic of the gRNA design for targeting the start codon of ANXA4 mRNA with dPspCas13b-NES.

(h) Representative distribution of ANXA4 immunohistochemistry (IHC) with the expression of the indicated PspCas13 variants and gRNAs. Typically, 3,000-8,000 cells that passed the expression threshold of mCherry fused to PspCas13 variants were considered.

(i) Quantification of ANXA4 IHC signals by FACS. The average values for the distributions (h) were used.

(j) Normalized ribosome footprint abundance (mean of 4 replicates). The genes examined in this study are highlighted. TPM, transcripts per kilobase million (considering footprints in the ORF).

In a-c, the mean (gray bar), s.d. (black line), and individual replicates ($n = 3$, black points) are shown.

In a, b, c, f, and i, p values were determined by Student’s t test (two-tailed) (b, c, f and i) and by the Tukey–Kramer test (two-tailed) (a).

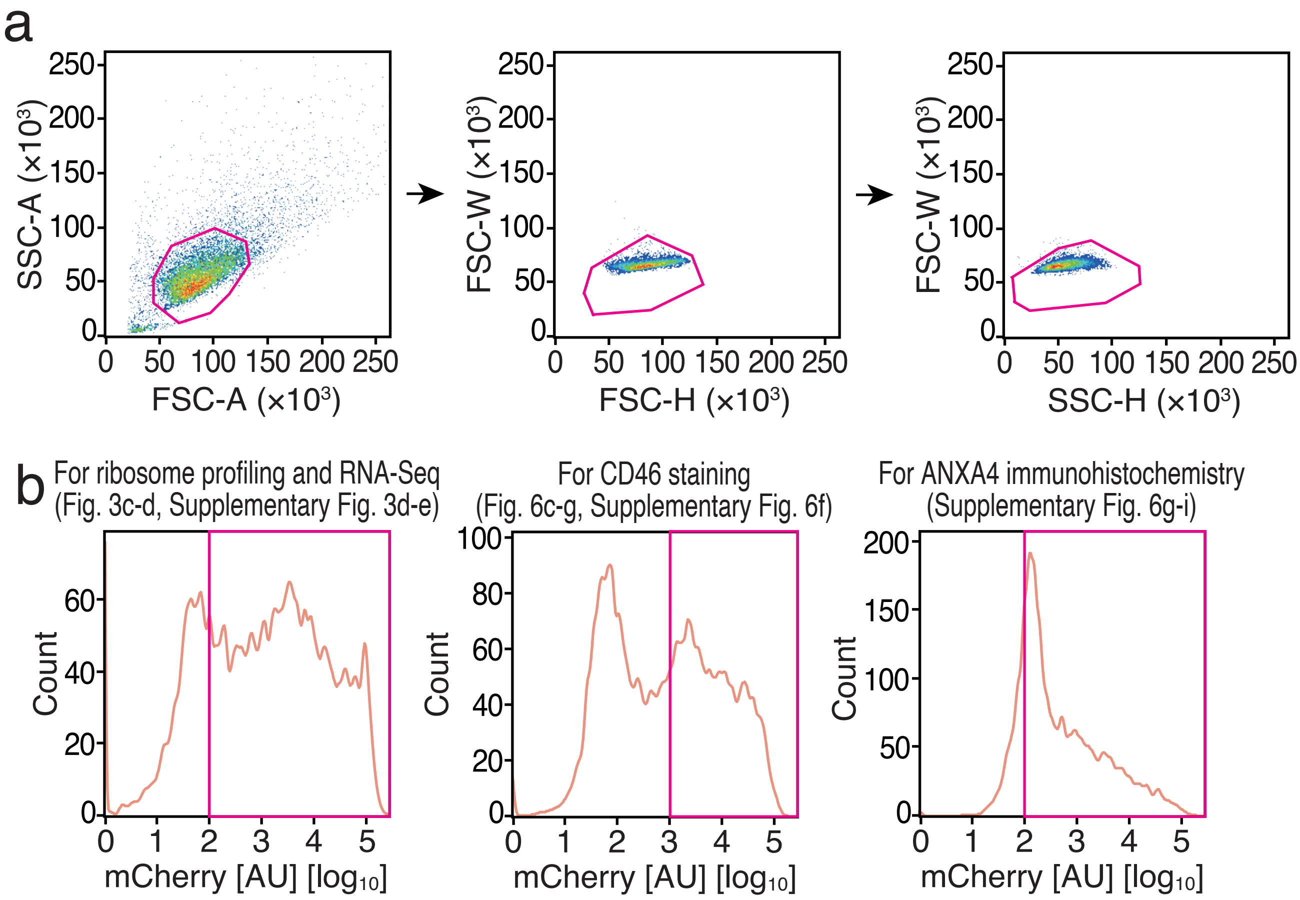
a
SSC-A (×10³)
FSC-A (×10³)
FSC-W (×10³)
FSC-H (×10³)
FSC-W (×10³)
SSC-H (×10³)
0
50
100
150
200
250
b
For ribosome profiling and RNA-Seq
(Fig. 3c-d, Supplementary Fig. 3d-e)
For CD46 staining
(Fig. 6c-g, Supplementary Fig. 6f)
For ANXA4 immunohistochemistry
(Supplementary Fig. 6g-i)
Count
mCherry [AU] [log10]
0
1
2
3
4
5
20
40
60
80
100
150
200

Supplementary Fig. 7: Gating strategy for FACS experiments.

(a) Representative gating strategy to exclude doublets and debris. This strategy was applied to all FACS experiments in this study.

(b) Gating strategy to select mCherry-positive cells. After removing doublets and debris, we excluded cells with less than these thresholds from the analysis.
