# Supplementary material for: dCas13-mediated translational repression for accurate gene silencing in mammalian cells
Source: Nat Commun. 2024 Mar 11;15:2205. doi: 10.1038/s41467-024-46412-7 (PMC10928199; doi:10.1038/s41467-024-46412-7)
Supplement: Supplementary file 2 — Description of Additional Supplementary Files [file 41467_2024_46412_MOESM2_ESM.pdf]

Supplementary Data 1: gRNA expression vectors prepared in this study.

Each plasmid is listed with the target, gRNA name, spacer sequence, DR sequence, and primer sets used for the construction.
