# Supplementary figures and images for: dCas13-mediated translational repression for accurate gene silencing in mammalian cells
Source: Nat Commun. 2024 Mar 11;15:2205. doi: 10.1038/s41467-024-46412-7 (PMC10928199; doi:10.1038/s41467-024-46412-7)

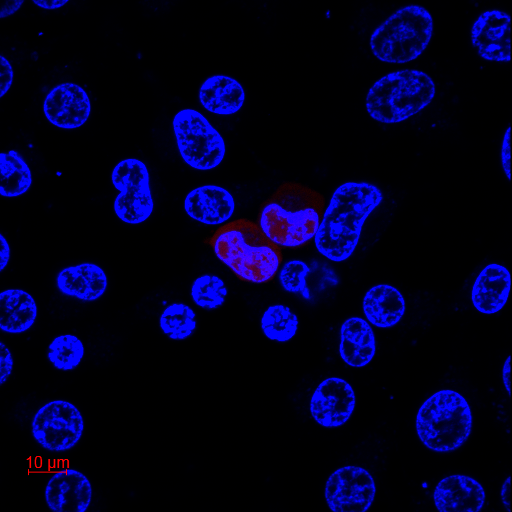

Supplement: Supplementary file 6 — Source Data [file 41467_2024_46412_MOESM6_ESM.zip › Source Data/SupplementaryFigure1i_NLS.tif]

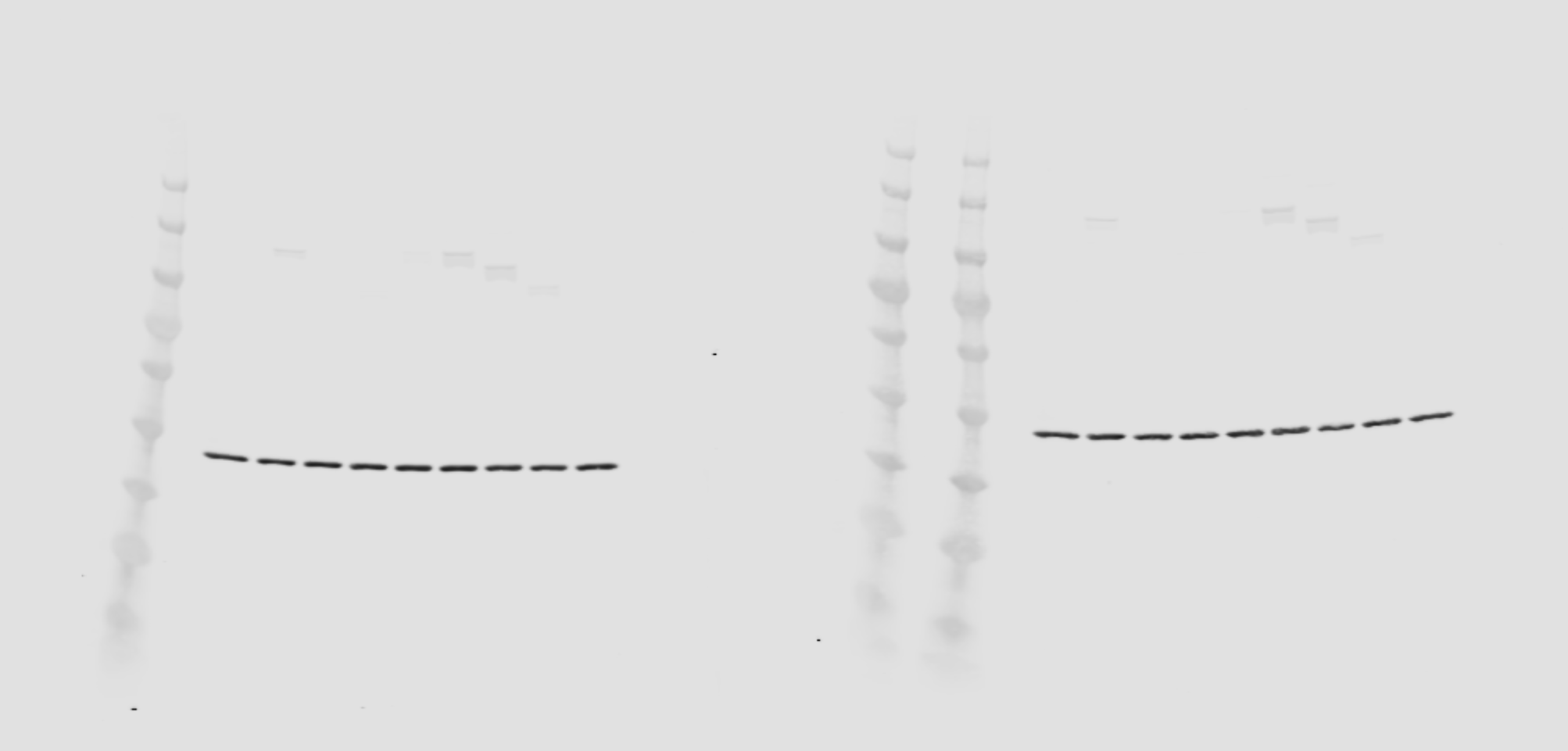

Supplement: Supplementary file 6 — Source Data [file 41467_2024_46412_MOESM6_ESM.zip › Source Data/SupplementaryFigure1c_Beta-Actin.tif]

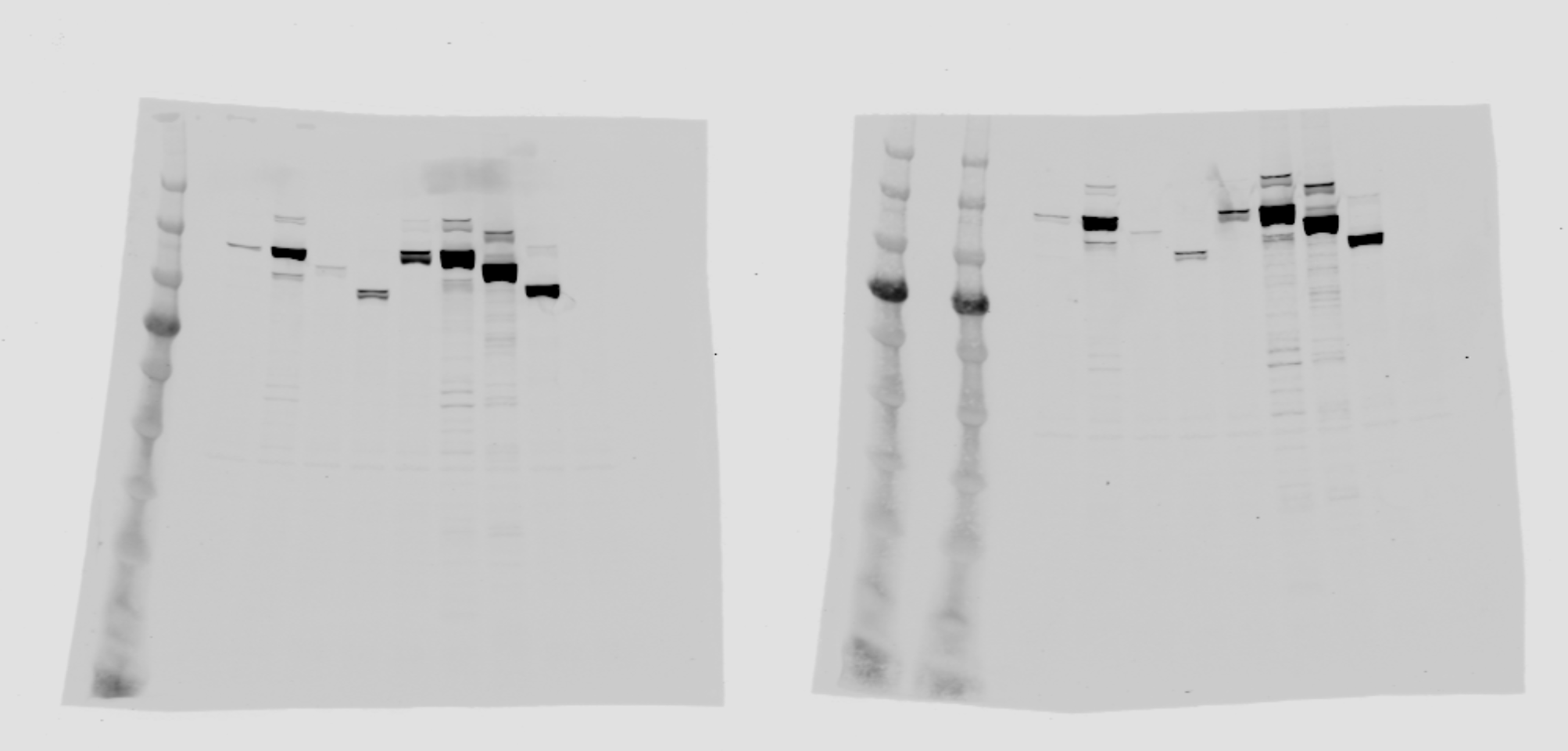

Supplement: Supplementary file 6 — Source Data [file 41467_2024_46412_MOESM6_ESM.zip › Source Data/SupplementaryFigure1c_Cas13_high_contrast.tif]

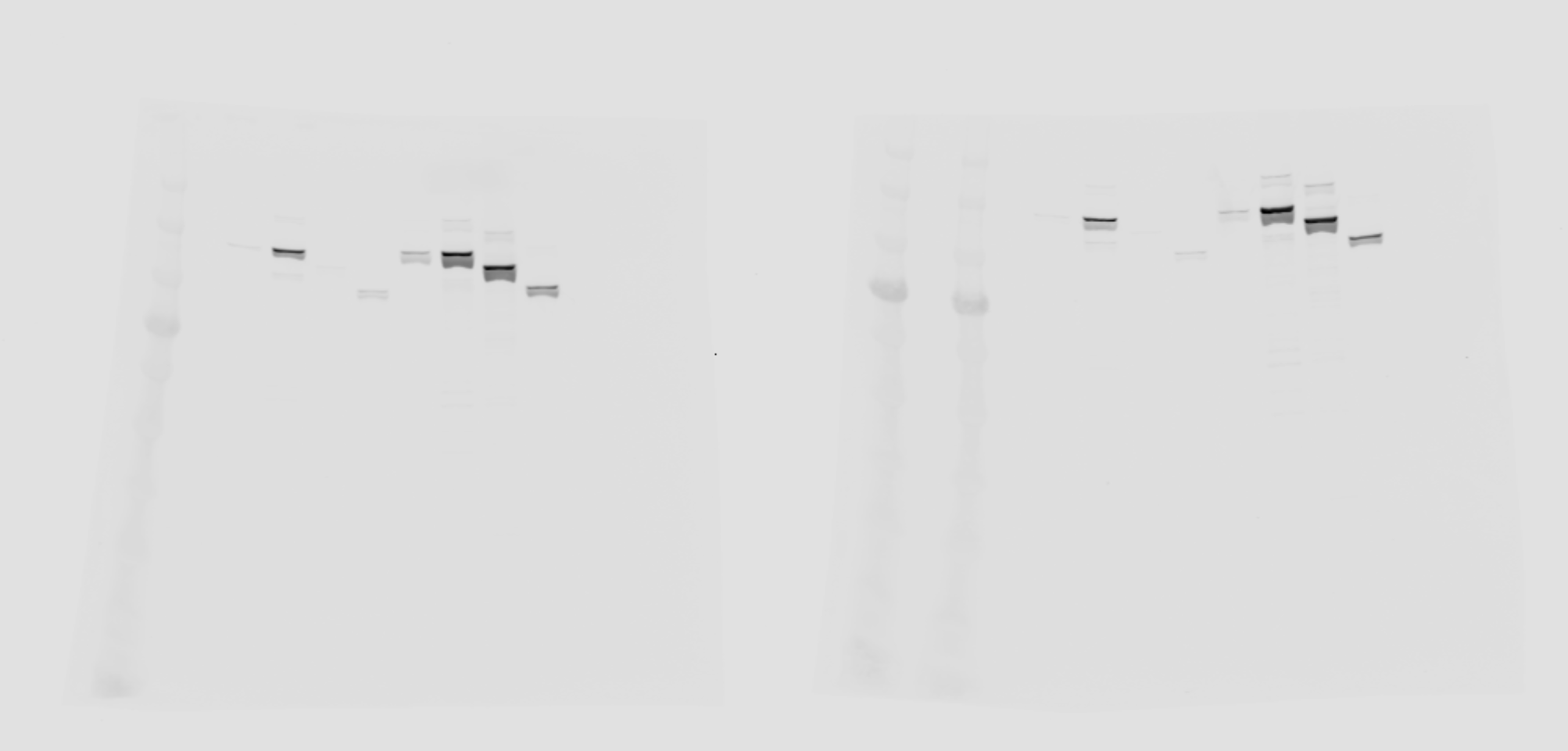

Supplement: Supplementary file 6 — Source Data [file 41467_2024_46412_MOESM6_ESM.zip › Source Data/SupplementaryFigure1c_Cas13_low_contrast.tif]

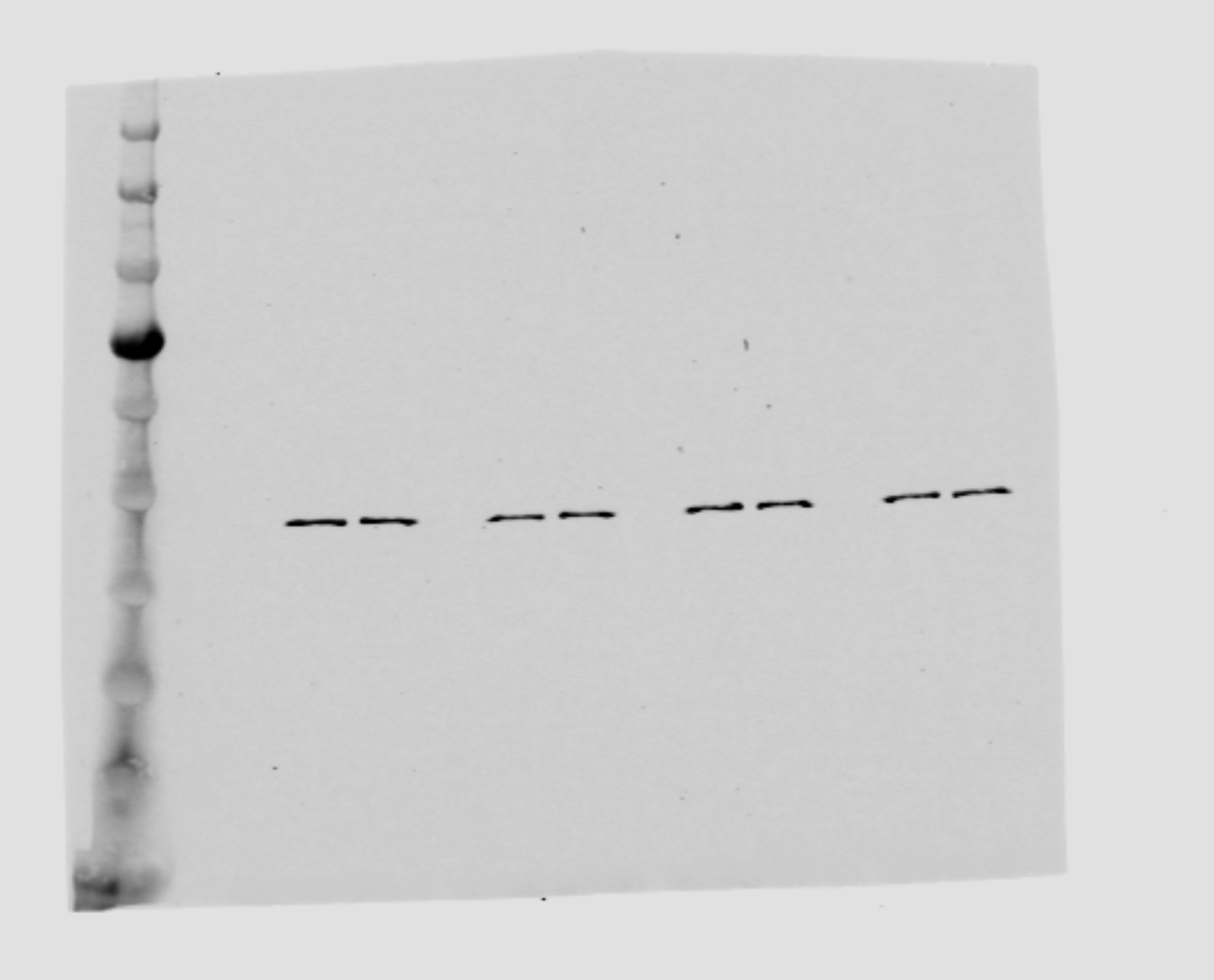

Supplement: Supplementary file 6 — Source Data [file 41467_2024_46412_MOESM6_ESM.zip › Source Data/Figure3e_betaActin.tif]

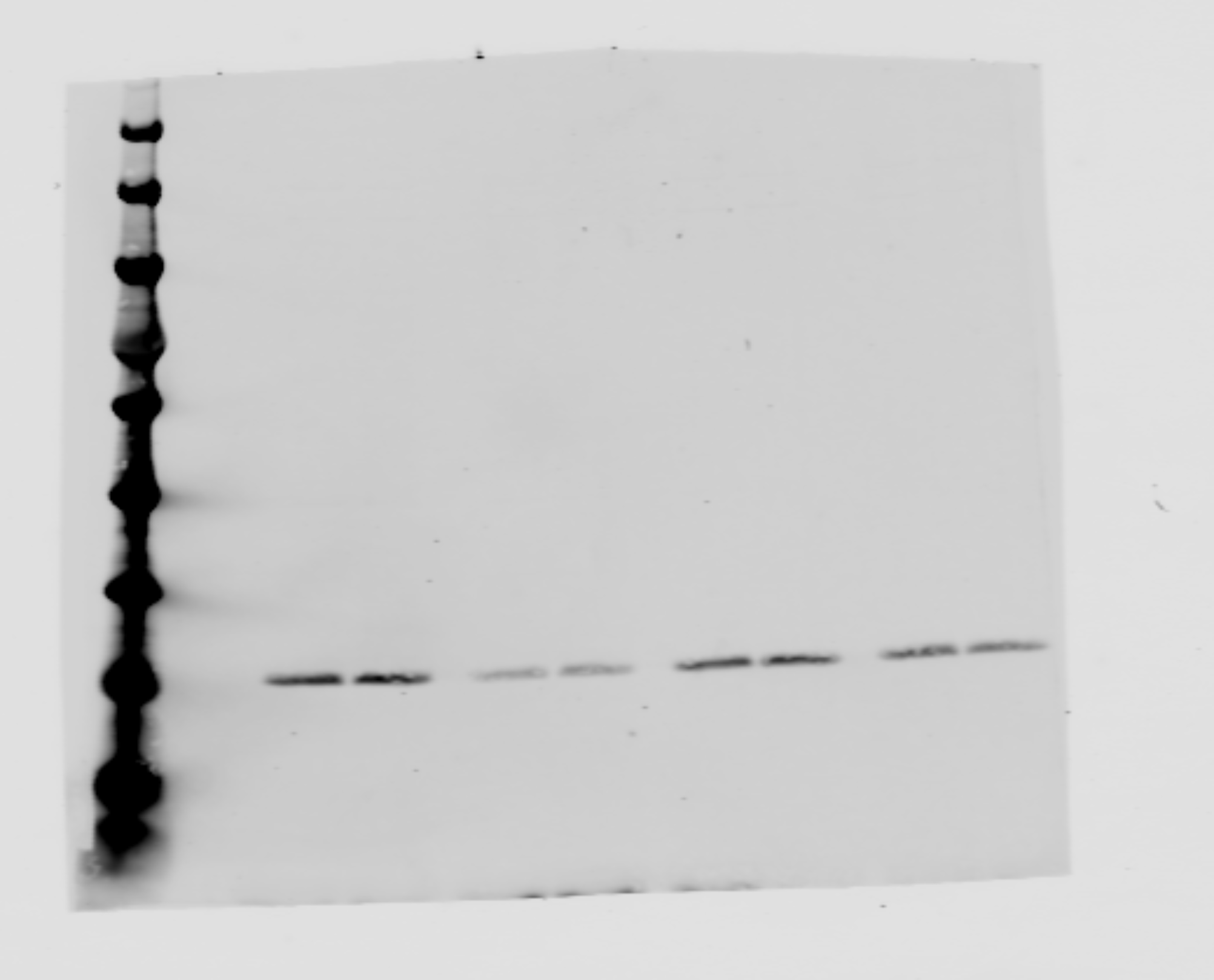

Supplement: Supplementary file 6 — Source Data [file 41467_2024_46412_MOESM6_ESM.zip › Source Data/Figure3e_EGFP.tif]

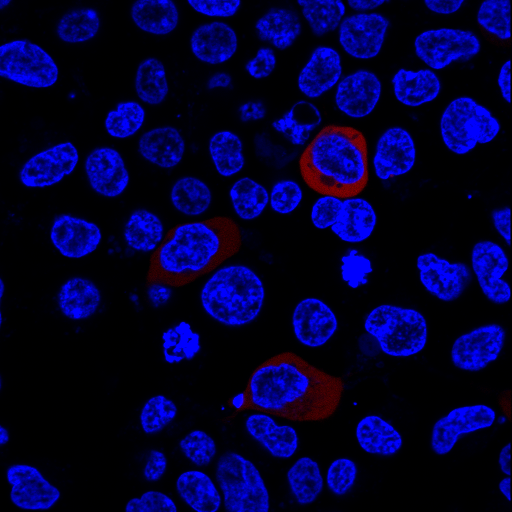

Supplement: Supplementary file 6 — Source Data [file 41467_2024_46412_MOESM6_ESM.zip › Source Data/SupplementaryFigure1i_NES.tif]
